# Comorbidities in psoriatic arthritis: a systematic review and meta-analysis

**DOI:** 10.1007/s00296-020-04775-2

**Published:** 2021-01-09

**Authors:** Sonal Gupta, Zoe Syrimi, David M. Hughes, Sizheng Steven Zhao

**Affiliations:** 1 School of Medicine, University of Liverpool, Liverpool, UK; 2 Department of Medicine, Liverpool University Hospitals, Liverpool, UK; 3 Department of Health Data Science, Institute of Population Health, University of Liverpool, Liverpool, UK; 4 Musculoskeletal Biology, Institute of Life Course and Medical Sciences, University of Liverpool, Liverpool, L69 3GA UK

**Keywords:** Psoriatic arthritis, Comorbidity, Multimorbidity, Systematic review, Meta-analysis

## Abstract

The aims of this systematic review and meta-analysis were to: (1) describe the prevalence of commonly reported comorbidities in psoriatic arthritis (PsA), (2) compare the incidence and/or prevalence of comorbidities between PsA and control populations; and (3) examine the impact of comorbidities on PsA outcomes. We systematically searched Medline, PubMed, Scopus, and Web of Science using a predefined protocol in accordance with PRISMA guidelines. Studies reporting only one comorbidity, or a few closely related diseases within one organ system, were excluded. Where possible, meta-analysis was performed using random-effects models. We included 39 studies amounting to over 152 thousand PsA patients. We performed meta-analysis for the prevalence of 21 commonly reported comorbidities. The most prevalent comorbidities were hypertension (pooled prevalence 34%), metabolic syndrome (29%), obesity (27%), hyperlipidaemia (24%) and any cardiovascular diseases (19%). Eleven studies consistently showed higher prevalence of comorbidities in PsA than controls. Five studies showed that comorbid patients had more severe disease, poorer quality of life, and increased discontinuation of treatment. Comorbidities, particularly cardiometabolic disorders, were highly prevalent in PsA and more common than in healthy controls. Comorbidities were associated with adverse disease features, but more research is needed on their impact on longitudinal outcomes such as treatment response, work productivity and mortality.

**Supplementary Information:**

The online version contains supplementary material available at 10.1007/s00296-020-04775-2.

## Introduction

Psoriatic arthritis (PsA) is a highly heterogeneous disease with numerous articular phenotypes and extra-articular disease features [1]. In addition, PsA patients commonly present with other coexisting medical conditions—comorbidities—or develop them after diagnosis. Comorbidities may be due to shared risk factors, consequences of reduced physical function and activity, chronic systemic inflammation and its treatment, or simply by chance. Studies in other chronic rheumatic diseases have shown that comorbidities are highly prevalent, and comorbidity burden is associated with poorer outcomes, such as quality of life, function and treatment response [2, 3]. They are also important considerations in routine clinical practice, by influencing treatment decisions (e.g. as contraindications). Others, such as cardiovascular diseases, are key drivers of mortality [4]. Despite the importance of comorbidities for clinical practice, a comprehensive approach to the study of comorbidities in PsA is lacking.

The aims of this systematic review and meta-analysis were to: (1) describe the prevalence of commonly reported comorbidities in PsA, (2) compare the incidence and/or prevalence of comorbidities between PsA and control populations; and (3) examine the impact of comorbidities on PsA outcomes.

## Methods

This systematic review was reported in accordance with the Preferred Reporting Items for Systematic Reviews and Meta-Analyses (PRISMA) guidelines [5]. The protocol for this review was pre-registered in advance (PROSPERO: CRD42020191047). We searched Medline, PubMed, Scopus, and Web of Science from inception to 24th of May 2020, using the following search term: psoriatic arthritis [MeSH] AND (multimorbid* OR comorbid* OR polymorbid* OR multi-morbid* OR co-morbid* OR poly-morbid* OR comorbidity [MeSH]).

Studies of PsA were included if they reported the prevalence or incidence of comorbidities or their impact on disease outcomes. Published abstracts were considered, but only if there was a sufficiently detailed description of study methodology and results. Studies were excluded if they focused on only one comorbidity of interest, or closely related diseases from one organ system (e.g. cardiovascular diseases only). This gives individual comorbidities context among other conditions, and distinguishes studies of comorbidity from, for example, cardiovascular risk. Furthermore, studies with risk of being non-representative of general PsA populations were excluded (e.g. males only or sample sizes < 30). Reviews, comments, and editorials were excluded. We also manually searched the bibliographies of all included papers to identify further eligible studies. Unpublished literature was not considered.

Titles and abstracts were screened by two independent reviewers (ZS and SG), who then assessed full-texts for inclusion and performed data extraction from eligible studies. Conflict at any stage was resolved through discussion moderated by a third reviewer (SSZ). We excluded psoriatic disease manifestations (skin involvement, enthesitis, dactylitis, and nail disease) and patho-mechanistically include conditions (uveitis and IBD) and inflammatory arthritides (given potential for misclassification/diagnosis) from our definition of comorbidities. Studies were assessed for risk of bias using adapted versions of the Newcastle Ottawa Scale (details in supplementary materials).

Where results for any comorbidity (in any of the three study aims) were reported by ≥ 3 studies, a meta-analysis was performed. Pooled prevalence estimates were reported as percentages (95% confidence interval, *I*^2^ statistic), using random-effects models (DerSimonian–Laird). Heterogeneity of meta-analysis estimates was presented using the *I*^2^ statistic. Funnel plots were used to assess risk of publication bias. Meta-analyses were performed using MetaXL Version 5.3 (Sunrise Beach, Australia).

## Results

**Fig. 1:**
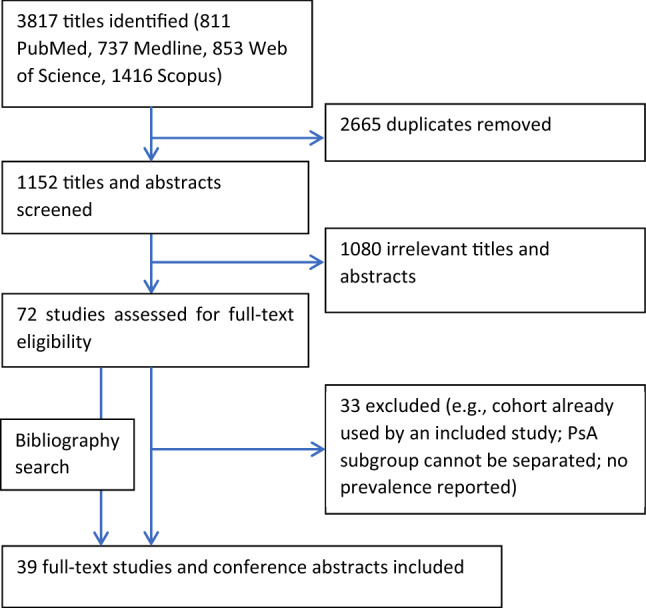
Study selection flowchart

A total of 3817 publications were returned by the literature search. After exclusions and de-duplication—shown in the Fig. 1 flowchart—39 studies remained. These studies are summarised in Supplementary Table S1. Sample sizes ranged from 32 to 35,061 with the total of 158,797 PsA patients. The selected studies comprised of 10 from the USA, 7 from the UK, 6 from Spain, 4 from Italy, 3 from Canada, 2 each from Denmark, Turkey, and France, and 1 each from Belgium, Brazil, Russia, the Netherlands, Romania, Israel, Taiwan and Hong Kong. Two studies recruited participants from multiple countries [6, 7].

PsA definition varied between studies, including CASPAR criteria (*n* = 11), Moll and Wright criteria (*n* = 2), self-report (*n* = 1), American College of Rheumatology definition (*n* = 1) or by physician diagnosis either from diagnostic codes (*n* = 15) or medical records (*n* = 4). Five studies did not report PsA definition. Comorbidities were defined by self-report confirmed by healthcare professional (*n* = 1), physician diagnosis either from diagnostic codes (*n* = 16) or medical records (*n* = 14); ascertainment was unclear in 8 publications. Most bias scores were 4 of a potential 6 stars (Supplementary Table S2 and Figure S1) indicating moderate bias. All studies did not justify their sample size thereby losing one star.

### Prevalence of comorbidities

A total of 39 studies reported prevalence of individual comorbidities with a combined sample size of 150,677 patients. The most frequently studied individual comorbidities were diabetes (*n* = 32 studies), hypertension (31) and hyperlipidaemia (18); all other were reported by 15 or fewer studies.

**Fig. 2:**
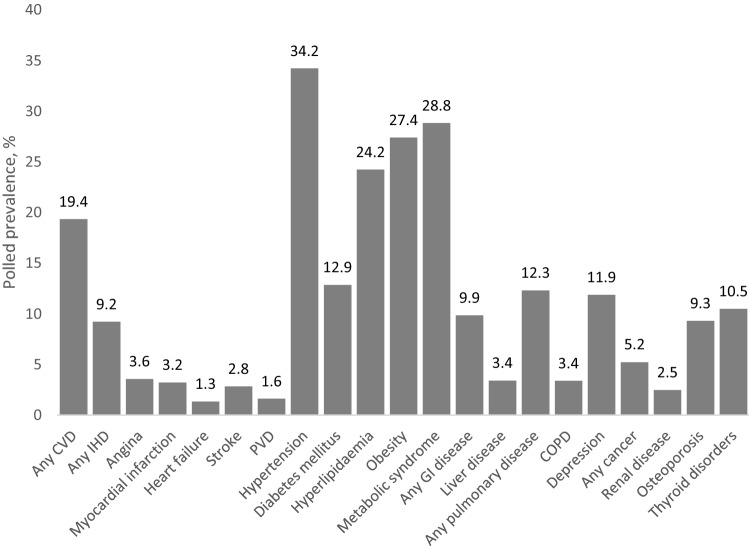
Pooled prevalence of comorbidities reported by ≥ 3 studies. *CVD* cardiovascular disease, *IHD* ischaemic heart disease, *PVD* peripheral vascular disease, *GI* gastrointestinal

Pooled prevalence estimates of individual comorbidities (reported by ≥ 3 studies) are summarised in Fig. 2 with further details in Table 1. The top five most prevalent comorbidities were hypertension (34.2%), metabolic syndrome (28.8%), obesity (27.4%), hyperlipidaemia (24.2%), and any CVD (19.4%). There was significant heterogeneity for most meta-analyses; stratification by PsA definition or comorbidity ascertainment did not improve heterogeneity (data not shown). Forest and funnel plots of the 21 meta-analyses are provided in supplementary materials.

**Table 1:** Meta-analysis estimates for prevalence of individual comorbidities

	No. of studies	No. of individuals	Pooled prevalence	95% confidence interval	*I*^2^, %	Range
Any CVD	12	44,369	19.4	7.8–34.1	100	3.1–70.5
Any IHD	8	29,671	9.2	7.1–11.6	97	0.6–18.2
Angina	3	5828	3.6	1.4–6.6	94	1.9–5.1
Myocardial infarction	10	17,628	3.2	2.3–4.2	87	1.3–8.1
Heart failure	8	23,455	1.3	1.0–1.7	72	0.6–3.1
Stroke	15	42,872	2.8	1.5–4.5	98	0.0–21.8
PVD	8	44,629	1.6	0.5–3.3	99	0.0–6.2
Hypertension	31	85,014	34.2	28.6–40.2	100	6.4–62.7
Diabetes mellitus	32	89,972	12.9	10.7–15.2	99	2.0–34.1
Hyperlipidaemia	18	59,816	24.2	17.4–31.8	100	2.9–79.8
Obesity	15	27,890	27.4	24.5–30.4	95	12.7–39.8
Metabolic syndrome	5	1109	28.8	14.0–46.2	96	9.9–44.0
Any GI disease	5	11,976	9.9	7.9–12.0	71	6.0–16.0
Liver disease	10	73,289	3.4	0.1–9.6	100	0.0–26.5
Any pulmonary disease	6	68,370	12.3	6.3–19.8	100	5.0–22.7
COPD	6	12,517	3.4	0–10.4	99	1.1–17.3
Depression	14	91,541	11.9	7.4–17.2	100	0.8–27.2
Any cancer	11	63,670	5.2	4.2–6.4	96	1.5–9.2
Renal disease	8	33,051	2.5	0.8–4.9	99	1.0–8.1
Osteoporosis	4	18,215	9.3	3.0–18.3	99	3.8–14.0
Thyroid disorders	5	25,316	10.5	7.7–13.7	94	0.6–15.0

Stroke includes cerebrovascular accidents and transient ischaemic attacks. *PVD* peripheral vascular disease, *COPD* chronic obstructive pulmonary disease, *GI* gastrointestinal

### Comorbidities in PsA compared to controls

Eleven studies compared comorbidities between PsA and control groups: one compared incidence, while the remaining reported prevalence (odds ratios, prevalence ratios, standardised mortality ratios). Most comorbidities were cardiometabolic disease or risk factors. All except three studies matched or adjusted for potential confounders such as age and sex. Virtually all individual comorbidities had higher incidence and prevalence in PsA populations than matched controls. The comorbidities and effect estimates were too heterogenous to permit meta-analysis.

Kaine et al. reported a 20–30% higher incidence of cardiovascular comorbidities (e.g. coronary, peripheral, or cerebrovascular diseases) in PsA vs matched controls, 44–67% higher incidence of mental health comorbidities, and over two-fold higher incidence of liver disease [8]. Prevalence of cardiovascular and mental health comorbidities was also consistently higher than controls. Effect estimates adjusted for confounders were smaller and less often significant; therefore, the three studies reporting unadjusted odds ratios had limited interpretation [9–11] (Table 2).

**Table 2:** Studies comparing comorbidity incidence between PsA patients and controls

	Measure of effect	Control group	Comorbidity	Effect size	95% confidence interval
Kaine 2019 [8]	Incidence rate ratio, matched by calendar year, age, gender and geography	Matched controls without PsA	Multiple sclerosis	**2.80**	1.85–4.24
			Hyperlipidaemia	**1.16**	1.11–1.21
			Hypertension	**1.21**	1.15–1.27
			Coronary artery disease	**1.25**	1.14–1.37
			Cerebrovascular disease	**1.31**	1.19–1.46
			Peripheral vascular disease	**1.31**	1.19–1.46
			Obesity or overweight	**1.58**	1.50–1.68
			Depression	**1.67**	1.57–1.78
			Anxiety	**1.44**	1.35–1.53
			Cancer	**1.09**	1.01–1.17
			Diabetes	**1.51**	1.40–1.64
			Osteoporosis	**1.78**	1.62–1.96
			Gout	**1.92**	1.72–2.15
			Liver disease	**2.15**	1.54–3.00
Cook 2018 [12]	SMR adjusted for age and sex	Matched UK biobank participants without AS, RA, PsA or SLE	Angina	**1.5**	1.1–1.7
			Myocardial infarction	1.3	0.8–1.9
			Stroke	1.0	0.6–1.6
			Hypertension	**1.4**	1.3–1.6
			Pulmonary disease	1.3	0.8–1.9
			Diabetes	1.2	0.9–1.6
			Depression	**1.3**	1.0–1.7
Haddad 2017 [13]	OR adjusted for variables including age, sex, smoking, obesity, and steroids use	Matched controls without rheumatic disease or psoriasis	Diabetes mellitus	**1.35**	1.18–1.42
			Hypothyroidism	**1.61**	1.47–1.81
			Osteoporosis	**1.56**	1.37–1.78
			Cushing disease	**3.96**	1.67–9.43
Feldman 2015 [14]	OR controlling for insurance type, individual non-PsO/PsA-related comorbidities, and modified Charlson Comorbidity Index	Matched controls without psoriasis and PsA	Hypertension	**1.9**	1.6–2.4
			Hyperlipidaemia	**1.3**	1.1–1.6
			Diabetes mellitus	**1.6**	1.2–2.2
			Coronary heart disease	**1.7**	1.1–2.5
			Acute myocardial infarction	0.5	0.1–3.0
			Anxiety	1.4	0.9–2.1
			Depression	**2.1**	1.5–3.0
			Obesity	1.4	0.8–2.3
			Cerebrovascular disease	1.5	0.7–3.6
			Peripheral vascular disease	1.4	0.7–2.8
			Skin cancer	1.5	0.7–3.1
			Other malignancies	0.8	0.4–1.7
			Multiple sclerosis	1.0	0.1–16.0
Han 2006 [15]	Prevalence ratio adjusted for age and sex	Matched controls from the same claims database	Ischaemic heart disease	**1.3**	1.1–1.5
			Atherosclerosis	**1.4**	1.0–2.1
			Congestive heart failure	**1.5**	1.1–2.0
			Peripheral vascular disease	**1.6**	1.2–2.0
			Cerebrovascular disease	**1.3**	1.1–1.7
			Type 2 diabetes	**1.5**	1.4–1.7
			Hyperlipidaemia	**1.2**	1.1–1.3
			Hypertension	**1.3**	1.2–1.4
Gladman 2008 [16]	Standardised Prevalence Ratio adjusted for gender and age	Age and sex matched standardisation population from community survey	Hypertension	**1.90**	1.59–2.27
			Cerebrovascular accident	0.91	0.34–2.43
			Myocardial infarction	**2.57**	1.73–3.80
			Angina	**1.97**	1.24–3.12
			Congestive heart failure	1.19	0.50–2.86
Jafri 2017 [17]	OR adjusted for age and sex	Matched controls from general UK primary care population	Hypertension	**1.31**	1.26–1.37
			Hyperlipidaemia	**1.23**	1.18–1.29
			Diabetes mellitus	**1.38**	1.31–1.45
			Obesity	**1.69**	1.62–1.75
Tam 2008 [18]	OR adjusted for BMI	Matched healthy controls	Hypertension	**3.37**	1.68–6.72
			Diabetes mellitus	**9.27**	2.09–41.09
Kristensen 2017 [9]	Unadjusted OR before diagnosis	Matched general population controls	Infections	**2.03**	1.69–2.42
			Neoplasms	**1.25**	1.11–1.41
			Haematological disorders	**1.94**	1.55–2.43
			Endocrine and metabolic disorders	**1.65**	1.48–1.84
			Mental disorders	1.15	0.97–1.36
			Nervous system disorders	**1.99**	1.75–2.26
			Cardiovascular disorders	**1.70**	1.56–1.86
			Respiratory disorders	**1.73**	1.54–1.96
			Digestive tract disorders	**1.89**	1.73–2.08
			Genitourinary disorder	1.33	0.75–1.04
	Unadjusted OR after diagnosis	Matched general population controls	Infections	**2.20**	1.89–2.55
			Neoplasms	**1.26**	1.14–1.40
			Haematological disorders	**2.13**	1.77–2.56
			Endocrine and metabolic disorders	**1.89**	1.72–2.07
			Mental disorders	**1.21**	1.14–1.40
			Nervous system disorders	**1.78**	1.58–2.00
			Cardiovascular disorders	**1.70**	1.57–1.85
			Respiratory disorders	**1.75**	1.57–1.95
			Digestive tract disorders	**1.98**	1.82–2.16
			Genitourinary disorder	**1.49**	1.36–1.63
Merola 2015 (abstract) [10]	Unadjusted OR	Matched controls from the same claims database	Chronic pulmonary disease	**1.73**	1.67–1.80
			Liver disease (excluding fatty liver)	**2.53**	2.39–2.67
			Anxiety	**1.53**	1.47–1.60
			Depression	**1.83**	1.76–1.91
			Coeliac disease	**2.51**	2.00–3.15
			Gout	**2.51**	2.33–2.71
Zhang 2011 (abstract) [11]	unadjusted OR	Matched controls without PsA	Hypertension	**1.58**	1.52–1.64
			Chronic pulmonary disease	**1.68**	1.59–1.77
			Diabetes	**1.62**	1.54–1.71
			Hypothyroidism	**1.64**	1.55–1.74
			Deficiency anaemias	**2.01**	1.88–2.14
			Depression	**1.69**	1.57–1.81
			Valvular diseases	**1.65**	1.53–1.78
			Psychoses	**1.76**	1.63–1.91
			Fluid electrolyte disorders	**1.81**	1.67–1.97
			Solid tumour without metastases	**1.17**	1.08–1.27
			Peripheral vascular disease	**1.72**	1.57–1.88

Results reported in the precision provided in publication. Bold text indicates statistical significance. *OR* odds ratio, *SMR* standardised mortality ratio, *BMI* body mass index, *PsO* psoriasis, *PsA* psoriatic arthritis

### Comorbidities and PsA outcomes

Five studies reported the impact of comorbidities on PsA disease outcomes (Table 3). In most studies, PsA patients with comorbidity had greater pain, functional limitation, and poorer quality of life than those without. Bavière et al. reported that the mental component score (MCS) of the Short Form 36 (SF-36) was significantly associated with the number of comorbidities [19]. For individual comorbidities, only anxiety was associated with MCS, while none were associated with the physical component score (PCS). Husted et al. found that only anxiety, depression and fibromyalgia were significantly associated with MCS [20], and only fibromyalgia and neurological disorders were associated with PCS. Only one paper reported the impact of comorbidities on treatment response. Stober et al. found that metabolic syndrome-related comorbidities were significantly associated with TNFi discontinuation (HR 2.65; *p* = 0.01) in multivariable models [21].

**Table 3:** Studies examining the effect of comorbidities on PsA disease outcomes

Study	How comorbidity was examined	Outcome	Results
Aydin 2016 (abstract) [22]	Patient reported	Tender joints Pain Patient global Assessment fatigue	Patients with at least one comorbidity had a higher disease activity than none: Tender joint count (3.9 vs. 2.9, *p* = 0.001), Pain (4.6 vs. 4.0, *p* = 0.006), Patient global assessment (4.5 vs. 4.0, *p* = 0.014), Fatigue (4.9 vs. 4.0, *p* < 0.001)
Bavière 2020 [19]	Modified RDCI derived from patient and physician report	SF-36 Physical (PCS) and Mental component scores (MCS)	In multivariable analysis of MCS, anxiety was strongly associated (*β* = − 10.81, *p* < 0.0001), however this was not seen for any other comorbidities MCS was significantly associated with the number of comorbidities (*β* = − 3.68, *p* < 0.0001) and mRDCI score (*β* = − 1.56, *p* = 0.0167)
Husted 2013 [20]	Self-report and medical records	SF-36 PCS and MCS	Patients with ≥ 3 comorbidities had lower (i.e. poorer) PCS (42.2 vs. 46.4) and MCS (46.4 vs. 48.5) than those with < 3 comorbidities In multivariable models, only fibromyalgia and neurological disorders were significantly associated with PCS (*p* < 0.001) while only fibromyalgia and anxiety/depression were associated with MCS
Fernandez-Carballido 2020 [23]	Medical records, to calculate CCI	HAQ	Patients with CCI > 1 had higher HAQ than CCI = 1 (median 0.75 vs. 0.25 *p* < 0.001) In multivariable analysis, HAQ was associated with: CCIp: *β* = 0.21 *p* < 0.001 Obesity: *β* = 0.19 *p* < 0.001 Hypertension: *β* = 0.20 *p* < 0.001 GI bleed: *β* = 0.49 *p* = 0.16 Hiatus hernia: *β* = 0.17 *p* = 0.07 Thyroid disease: *β* = 0.18 *p* = 0.06
Stober 2018 [21]	Medical records	Discontinuation of the first TNF inhibitor	In multivariable models, metabolic syndrome-related comorbidities were independently significantly associated with TNFi discontinuation (HR 2.65; *p* = 0.01)

*PsA* psoriatic arthritis, *TNFI* tumour necrosis factor inhibitor, *HR* hazard ratio, *FM* fibromyalgia, *SF-36* Short Form-36, *PCS* Physical component score, *MCS* Mental component scores, *CCIp* Charlson Comorbidity Index proxy, *mRDCI* modified Rheumatic Disease Comorbidity Index (mRDCI), *HAQ* Health Assessment Questionnaire

## Discussion

This meta-analysis combining data from over 150 thousand PsA patients showed that comorbidities, particularly cardiometabolic disorders, are highly prevalent, with around 1 in 3 having hypertension, 3 in 10 having metabolic syndrome, and 1 in 4 having obesity. Almost all comorbidities were more common in PsA patients than controls. The presence and number of comorbidities were associated with poorer quality of life, function, and discontinuation of TNF inhibitors.

Our results showed a clear predilection for cardiometabolic comorbidities in PsA. The top five most prevalent comorbidities—hypertension (34%), metabolic syndrome (29%), obesity (27%), hyperlipidaemia (24%) and overall cardiovascular diseases (CVD; 19%)—provide an interesting comparison against comorbidity patterns in axSpA (another member of the SpA family). In a similar review of comorbidities in axSpA [24], the commonest comorbidities were similar but differed in prevalence (hypertension (23%), infections (18%), hyperlipidaemia (17%), obesity (14%) and CVD (12%)). For example, obesity was nearly twice more common in PsA than axSpA. Although patho-mechanistically related, there are clearly different disease mechanisms at play in these two SpA phenotypes that drive comorbidity patterns. Higher prevalence of cardiometabolic disorders may partly be explained by the greater systemic inflammatory burden in peripheral joint involvement, but also the unique pathology in skin disease. Chronic psoriasis generates vascular endothelial growth factor (VEGF) and oxidative stress that directly contribute to cardiometabolic derangements [25]. Together, these findings highlight the need for better CVD risk assessment and stratification in PsA and other chronic rheumatic diseases, to prevent or reduce cardiovascular morbidity and mortality. Interestingly, despite differences in cardiometabolic risk factors, the prevalence of cardiovascular diseases was similar between PsA and axSpA: any CVD 19 vs. 12%; angina 3.6 vs. 3.6%; myocardial infarction 3.2 vs. 2.2%; heart failure 1.3 vs. 1.8%; stroke 2.8 vs. 1.8%; peripheral vascular disease 1.6 vs. 1.1%, respectively [24].

This review also showed high prevalence of pulmonary diseases and depression (each 12%), both of which are more common than in controls. Depression is a well-recognised comorbidity in psoriatic diseases that is likely underdiagnosed. Prevalence in this review is lower than reported elsewhere when using screening tools [26]. Improved screening and optimisation of mental health comorbidities are essential as they can influence patients’ experience of symptoms and treatment adherence [26]. The links between PsA and pulmonary comorbidities are receiving increasing research focus. Several observational studies have suggested a causal link between metabolic syndrome and lung disease, for example, through obesity hypoventilation and obstructive sleep apnoea [27]. Equally, they may both arise from shared risk factors such as smoking, which is causally associated with chronic obstructive pulmonary disease and PsA [28, 29].

The number of studies that assessed the impact of comorbidities on PsA outcomes was relatively scarce. Most showed cross-sectional associations between comorbidity burden and reduced quality of life, particularly the MCS (mental component score) of the SF-36. We found no studies of whether comorbidities influenced treatment response or longer-term outcomes such as work productivity or mortality. These represent urgent unmet research needs to quantify the individual and societal impact of comorbidities in PsA.

A strength of this review is the broad inclusion of comorbidities. This approach provides wider context for the epidemiology and impact of individual diseases. However, comorbidity ascertainment may well be different in PsA than controls. People with chronic diseases are likely to have more frequent contact with healthcare providers thus opportunities to screen for and/or diagnose comorbidities. For example, hypothyroidism was more prevalent in PsA than controls in two studies—there is limited biological rationale for why there should be higher prevalence in PsA. This may explain the greater comorbidity burden across almost all comorbid conditions. However, there is also opposing evidence that suggest poorer identification and management of comorbidities in people with rheumatic diseases than without [30]. Prevalence estimates may be influenced by the fact that we did not include studies reporting individual comorbidities (e.g. depression in PsA). This was decided a priori for three reasons. First, such studies typically use more sensitive methods of ascertainment (e.g. screening questionnaires) thus may bias true estimates and introduce additional heterogeneity. Second, our objective was to review how common comorbidities were studied collectively. Focusing on depression without considering, for example, the co-existence of fibromyalgia or heart disease would be contrary to the aim of comorbidities research set to inform a holistic management approach. Third, systematically reviewing and discussing each individual condition is beyond the scope of one standalone paper. Another limitation was the high heterogeneity in meta-analysis estimates. This partly reflects the diverse case definition for PsA and comorbidities (though stratifying results did not improve heterogeneity), but also differences in study population, disease duration and treatment. Most included studies were cross-sectional in design; therefore, we were not able examine chronology of comorbidity development, though the study by Kristensen et al. suggested prevalence to be similar before and after diagnosis of PsA [9]. Future studies should investigate whether treatments contribute to (e.g. NSAIDs on CVD) or prevent (e.g. pain control and improvement in mental health) comorbidities development in PsA.

In conclusion, this systematic review showed that comorbidities are highly prevalent among patients with PsA, particularly cardiometabolic, but also mental health and pulmonary diseases. Comorbidities were more common in PsA patients than controls, and were associated with poorer quality of life, function, and discontinuation of TNF inhibitors. Research on the impact of comorbidities on longitudinal outcomes is needed, including treatment response, work productivity and mortality.

## Supplementary Information

Below is the link to the electronic supplementary material.

Supplementary file1 (DOCX 482 KB)
