# Supplementary material for: Comorbidities in psoriatic arthritis: a systematic review and meta-analysis
Source: Rheumatol Int. 2021 Jan 9;41(2):275–84. doi: 10.1007/s00296-020-04775-2 (PMC7835184; doi:10.1007/s00296-020-04775-2)
Supplement: Supplementary file 1 — Supplementary file1 (DOCX 482 KB) [file 296_2020_4775_MOESM1_ESM.docx]

Supplementary materials for

“**Comorbidities in psoriatic arthritis: a systematic review and meta-analysis**”

Sonal Gupta, Zoe Syrimi, David Hughes, Sizheng Zhao

| Supplementary Table S1. Summary of studies included in the systematic review of comorbidities in PsA | | | | | | | | |  |
| --- | --- | --- | --- | --- | --- | --- | --- | --- | --- |
| Study | Country | Data Source | Sample Size | PsA Diagnosis | Mean Age | Male, % | Comorbidity Diagnosis | Number of Comorbidities | |
| ABSTRACT Aydin 2016 | Turkey | Psoriatic Arthritis Registry of Turkey | 1069 | NA | 46.8 | 35 | NA | 11 | |
| ABSTRACT Batkaeva 2016 | Russia | Dermatology hospital cohort | 220 | Medical records and ICD | 53.3 | NA | Medical records and ICD | 3 | |
| ABSTRACT Generali 2016 | Italy | Administrative health databases | 8981 | NA | Median: 51.9 | 50 | NA | 7 | |
| ABSTRACT Gudu 2017 | Romania | Outpatients | 129 | Physician diagnosed | 53.5 | 40 | Physician diagnosed | 14 | |
| ABSTRACT Gurcay 2007 | Turkey | NA | 32 | American College of Rheumatology PsA Definition | 46.2 | NA | NA | 10 | |
| ABSTRACT Merola 2015 | USA | Truven claims database | 35,061 | ICD | 49.1 | 47 | ICD | 8 | |
| ABSTRACT Pazmino 2019 | The Netherlands | GP-based morbidity registration network | 190 | International Classification of Primary Care codes | 47 | 52 | International Classification of Primary Care codes | 8 | |
| ABSTRACT Zhang 2011 | USA | US administrative claims database | 21332 | ICD | 52 | 47 | ICD | 13 | |
| Bavière 2020 | France | Multicentre Outpatients | 124 | CASPAR | 52.6 | 62 | Self-report/ physician report | 9 | |
| Christophers 2009 | Multiple | Multicentre Dermatology Outpatients | 126 | Physician diagnosed | 49.1 | 68 | Physician diagnosed | 8 | |
| Cook 2018 | UK | UK Biobank | 865 | ICD | 57 | 49 | ICD | 7 | |
| Cooksey 2018 | UK | Welsh GP Database | 2128 | ICD | 38.5 | 32 | ICD | 4 | |
| Costa 2016 | Italy | Rheumatology outpatients | 618 | CASPAR | 52.1 | 39 | ICD | 8 | |
| Dai 2018 | Taiwan | National Health Insurance Research Database | 8795 | ICD | 44.5 | 60 | ICD | 10 | |
| Dalal 2015 | USA | Hospital outpatients | 107 | Physician diagnosed confirmed by CASPAR | 51 | 58 | Physician diagnosed | 4 | |
| Edson-Heredia 2015 | UK | UK primary care | 1952 | Physician diagnosed | 49 | 49 | Physician diagnosed | 11 | |
| Favarato 2014 | Brazil & USA | Electronic charts registries | 158 | CASPAR | 53.7 | 47 | Physician diagnosed | 3 | |
| Feldman 2015 | USA | Health insurance claims database | 1230 | ICD | 48.46 | 52 | ICD | 26 | |
| Fernández-Carballido 2020 | Spain | Cardiovascular in Rheumatology (CARMA) project | 721 | Moll and Wright criteria | NA | NA | NA | 18 | |
| Gladman 2008 | Canada | Hospital outpatients | 648 | Physician Diagnosed | 43.5 | 56 | Physician diagnosed | 6 | |
| Gonzalez-Martin 2019 | Spain | Hospital outpatients | 76 | NA | 55.8 | 54 | NA | 7 | |
| Haddad 2017 | Israel | Hospital outpatients | 3161 | NA | 58.4 | 47 | Medical records | 5 | |
| Han 2006 | USA | Claims database | 3066 | ICD | 49.7 | 27 | ICD | 8 | |
| Haque 2015 | Belgium | Spondyloarthritis Registry (SPAR) | 262 | CASPAR | 58.8 | 60 | Medical records | 10 | |
| Højgaard 2018 | Denmark | DANBIO register and the Danish National Patient Register | 1750 | ICD | M 46.9, F 48.8 | 47 | ICD | 6 | |
| Husted 2013 | Canada | Specialist and community clinic outpatients | 631 | CASPAR | 49.60% | 59 | Medical records | 15 | |
| Jafri 2017 | USA / UK | The Health Improvement Network (THIN) | 12548 | Diagnostic codes | 50.07 | 63 | Diagnostic codes | 5 | |
| Kaine 2009 | USA | Claims Databases | 16,248 | ICD | 53.4 | 45 | ICD | 14 | |
| Kavanaugh 2018 | Multiple | Hospital outpatients | 4315 | Self-report | 50.4 | 51 | By HCP and self-report | 15 | |
| Kristensen 2017 | Denmark | Danish Patient Registry | 10525 | ICD | 52 (median) | 41 | ICD | 12 | |
| Nas 2015 | Turkey | Hospital outpatients | 173 | CASPAR | 41.8 | 43 | Physician diagnosed | 7 | |
| Ogdie 2015 | UK | Primary care medical record database in the UK | 8706 | Diagnostic codes | 50-52 | 49 | Diagnostic codes | 3 | |
| Ortolan 2019 | Italy | Hospital outpatients | 43 | CASPAR | 60.2 | 74 | Physician diagnosed | 4 | |
| Puig 2013 | Spain | NA | 84 | NA | 42.8 | 69 | NA | 4 | |
| Queiro 2018 | Spain | Hospital outpatients | 340 | CASPAR | 55 | 56 | NA | 4 | |
| Queiro 2020 | Spain | Hospital outpatients | 223 | CASPAR | 54.5 | 5 | NA | 4 | |
| Stober 2018 | UK | Electronic medical records | 188 | CASPAR | 47.2 | 49 | Physician diagnosed | 4 | |
| Tam 2008 | Hong Kong | Hospital records | 102 | Moll and Wright criteria | 48.7 | 49 | Medical records | 4 | |
| Wu 2017 | USA | Claims database | 5138 | ICD | 54 | 48 | ICD | 4 | |

| Supplementary Table S2. Quality assessment using a modified Newcastle-Ottawa Scale. | | | | | |
| --- | --- | --- | --- | --- | --- |
| Study | Representativeness | Sample Size | PsA definition | Ascertainment of comorbidities | Total |
| ABSTRACT Aydin 2016 | 1 | 0 | 2 | 1 | 4 |
| ABSTRACT Batkaeva 2016 | 0 | 0 | 1 | 1 | 2 |
| ABSTRACT Generali 2016 | 1 | 0 | 1 | 1 | 3 |
| ABSTRACT Gudu 2017 | 1 | 0 | 2 | 0 | 3 |
| ABSTRACT Gurcay 2007 | 1 | 0 | 2 | 1 | 4 |
| ABSTRACT Merola 2015 | 2 | 0 | 2 | 0 | 4 |
| ABSTRACT Pazmino 2019 | 2 | 0 | 1 | 1 | 4 |
| ABSTRACT Zhang 2011 | 2 | 0 | 1 | 1 | 4 |
| Bavière 2020 | 2 | 0 | 2 | 1 | 5 |
| Christophers 2009 | 1 | 0 | 0 | 1 | 2 |
| Cook 2018 | 2 | 0 | 0 | 1 | 3 |
| Cooksey 2018 | 2 | 0 | 1 | 1 | 4 |
| Costa 2016 | 1 | 0 | 2 | 1 | 4 |
| Dai 2018 | 2 | 0 | 2 | 1 | 5 |
| Dalal 2015 | 1 | 0 | 2 | 1 | 4 |
| Edson-Heredia 2015 | 2 | 0 | 1 | 1 | 4 |
| Favarato 2014 | 1 | 0 | 2 | 0 | 3 |
| Feldman 2015 | 2 | 0 | 2 | 1 | 5 |
| Fernández-Carballido 2020 | 1 | 0 | 2 | 1 | 4 |
| Gladman 2008 | 1 | 0 | 2 | 1 | 4 |
| Gonzalez-Martin 2019 | 1 | 0 | 0 | 1 | 2 |
| Hadad 2017 | 2 | 0 | 2 | 1 | 5 |
| Han 2006 | 2 | 0 | 2 | 1 | 5 |
| Haque 2015 | 1 | 0 | 2 | 1 | 4 |
| Højgaard 2018 | 0 | 0 | 1 | 1 | 2 |
| Husted 2013 | 1 | 0 | 2 | 1 | 4 |
| Jafri 2017 | 2 | 0 | 1 | 1 | 4 |
| Kaine 2009 | 2 | 0 | 1 | 0 | 3 |
| Kavanaugh 2018 | 1 | 0 | 0 | 0 | 1 |
| Kristensen 2017 | 2 | 0 | 1 | 1 | 4 |
| Nas 2015 | 1 | 0 | 2 | 1 | 4 |
| Ogdie 2015 | 2 | 0 | 1 | 1 | 4 |
| Ortolan 2019 | 1 | 0 | 2 | 1 | 4 |
| Puig 2013 | 0 | 0 | 0 | 1 | 1 |
| Queiro 2018 | 1 | 0 | 2 | 1 | 4 |
| Queiro 2020 | 1 | 0 | 2 | 0 | 3 |
| Stober 2018 | 0 | 0 | 2 | 0 | 2 |
| Tam 2008 | 2 | 0 | 2 | 1 | 5 |
| Wu 2017 | 2 | 0 | 2 | 1 | 5 |
| Representativeness: 2 for population or primary care level data, 1 hospital, 0 more selective (eg for biologic DMARD treatment). Sample size: 1 if justified, 0 if not. PsA definition (“exposure”): 2 for classification criteria or physician diagnosis; 1 for diagnostic codes; 0 if self-reported. Ascertainment of comorbidities (“outcome”): 1 if diagnostic code; 0 if self-reported. 0 is given if unclear or unreported. | | | | | |

Figure S1. Distribution of NOS bias scores from Table S2.

Forest and funnel plots for each meta-analysis

Any CVD

Any IHD

Angina

Myocardial infarction

Heart failure

Stroke

Peripheral vascular disease

Hypertension

Diabetes mellitus

Hyperlipidaemia

Obesity

Metabolic syndrome

Any gastrointestinal disease

Liver disease

Any pulmonary disease

COPD

Depression

Any cancer

Renal disease

Osteoporosis

Thyroid disorders
